# A Bayesian Account of the Sensory-Motor Interactions Underlying Symptoms of Tourette Syndrome

**DOI:** 10.3389/fpsyt.2019.00029

**Published:** 2019-03-05

**Authors:** Charlotte L. Rae, Hugo D. Critchley, Anil K. Seth

**Affiliations:** ^1^Sackler Centre for Consciousness Science, University of Sussex, Brighton, United Kingdom; ^2^Department of Neuroscience, Brighton and Sussex Medical School, Brighton, United Kingdom; ^3^Sussex Partnership NHS Foundation Trust, Brighton, United Kingdom; ^4^School of Engineering and Informatics, University of Sussex, Brighton, United Kingdom

**Keywords:** active inference, basal ganglia, insula, motor cortex, tics, Tourette syndrome

## Abstract

Tourette syndrome is a hyperkinetic movement disorder. Characteristic features include tics, recurrent movements that are experienced as compulsive and “unwilled”; uncomfortable premonitory sensations that resolve through tic release; and often, the ability to suppress tics temporarily. We demonstrate how these symptoms and features can be understood in terms of aberrant predictive (Bayesian) processing in hierarchical neural systems, explaining specifically: why tics arise, their “unvoluntary” nature, how premonitory sensations emerge, and why tic suppression works—sometimes. In our model, premonitory sensations and tics are generated through over-precise priors for sensation and action within somatomotor regions of the striatum. Abnormally high precision of priors arises through the dysfunctional synaptic integration of cortical inputs. These priors for sensation and action are projected into primary sensory and motor areas, triggering premonitory sensations and tics, which in turn elicit prediction errors for unexpected feelings and movements. We propose experimental paradigms to validate this Bayesian account of tics. Our model integrates behavioural, neuroimaging, and computational approaches to provide mechanistic insight into the pathophysiological basis of Tourette syndrome.

## Introduction

Tourette syndrome (TS) remains an enigmatic disorder. It is a chronic neuropsychiatric condition of neurodevelopmental origin. The likely pathoaetiology of TS is a combination of (epi)genetic influences, dysconnectivity within cortico-striato-thalamo-cortical (CSTC) circuits, and neurochemical alterations. Clinically TS is characterised by the recurrent expression of tics, which are often preceded by premonitory sensations or urges, and are under partial volitional control.

Here, we detail how these primary symptoms and features of TS can be accommodated within a neuroanatomically explicit framework of action-oriented predictive processing—or active inference (1, 2). In this framework, the brain continuously engages in the minimisation of sensory “prediction errors,” i.e., mismatches between sensory signals and prior expectations, by updating perceptual priors and by performing actions to change sensory signals, through approximations to Bayesian inference. This model makes clear predictions for behavioural measures of voluntary action in TS, tied to specific neuroanatomical networks. We review evidence for this Bayesian account of tics, and propose experimental approaches to test the implications of this model.

## Features of Tourette Syndrome

TS presents a multifaceted set of phenomenological features (3) (Table 1). Tics, the most obvious and distinguishing symptoms, are typically brief actions or vocalisations, which range in complexity from simple recurrent acts such as eye blinks or coughs, to elaborate action sequences. Tourette syndrome is defined by the diversity of tics (i.e., the presence of multiple motor tics, and at least one phonic tic), chronicity (persistence over months across the lifespan) and neurodevelopmental origin (childhood onset). Individuals presenting with isolated motor or phonic tics only are described as having a persistent tic disorder. While these diagnostic criteria indicate the greater complexity of Tourette syndrome, many consider the diagnostic boundary to lie along a spectrum of tic disorder severity (28).

**Table 1:** Features of Tourette syndrome.

ADHD (Attention Deficit Hyperactivity Disorder)	Neurodevelopmental condition commonly comorbid in TS, at rates of up to 66% (4). Notably, there is overlap with TS in broad dysfunction of subcortical circuitry and interactions with prefrontal cortex
Cortico-striato-thalamo-cortical (CSTC) circuits	These pathways are proposed as core substrates of dysfunction in TS: aberrant processing of cortical inputs to basal ganglia can engender dysfunctional inhibitory striatal output. Detailed knowledge of the nature of CSTC circuit perturbation is still at an early stage of understanding
Developmental onset	Tics typically emerge in childhood or adolescence (3). The developmental course of TS varies; many people with TS experience a gradual remission of tics through adolescence into adulthood (5). Tics may also (re-)emerge later in life, often, though not always, during periods of psychological stress
Direct and indirect pathways	Two pathways through the basal ganglia, in which activity either promotes movement (direct pathway) or withholds movement (indirect pathway). The sequence of excitatory and inhibitory connections within the pathways leads to either disinhibition of thalamic signals to the primary motor cortex, and release of action (direct pathway) or maintenance of inhibitory thalamic signals, and prevention of action (indirect pathway) (6). Neuromodulators such as dopamine weight synaptic interactions within the pathways, balancing their influence over thalamic output
Dopamine	Dopamine is implicated in TS, not least because drugs that constrain dopaminergic D2 activation (including sulpiride, aripiprazole, pimozide, and risperidone) can reduce tics (7). These remain the most commonly prescribed medications for TS, although it is noteworthy that they do not effectively reduce tics in all individuals (8). Dopaminergic therapies may reduce tics by dampening facilitation of movement through modulation of glutamatergic and GABAergic connections that stabilise interactions between the direct and indirect pathways. The role of dopamine in TS remains controversial; various unconfirmed proposals include hyper-innervation, oversensitive receptors, pre-synaptic abnormalities, and tonic-phasic imbalance (9)
GABA	Disrupted GABAergic transmission is increasingly recognised as a candidate mechanism in the pathophysiology of TS; highlighted by post-mortem data indicating reduced numbers of striatal GABAergic interneurons (10–12) and neuroimaging evidence for perturbation in GABA levels across multiple cortical sites (13, 14)
Glutamate	Alongside disruptions to GABAergic function, alterations in glutamatergic signalling likely contribute to proposed excitatory and inhibitory imbalances within CSTC circuitry (15–17)
Morphological alterations	Structural imaging studies of TS highlight abnormalities in grey matter volume and white matter architecture within CSTC circuits, involving lateral prefrontal, cingulate and primary somatosensory cortices, putamen, caudate nucleus, thalamus, and connecting tracts (18, 19). Longitudinal studies to index maturational trajectories of these differences remain scarce (20)
Neurochemical disruptions	Beyond GABA, glutamate, and dopamine transmission, a wider expression of neurochemical disruptions may contribute to pathophysiological differences in TS, including dysfunctional noradrenergic, serotonergic, cholinergic, histamine, and cannabinoid systems (15, 17, 20). Correspondingly, many of these neuromodulatory systems influence cortico-subcortical interactions
OCD (Obsessive Compulsive Disorder)	Commonly comorbid in TS, at rates of up to 35% (4). Obsessive compulsive symptoms typically emerge a few years after the onset of tics. There is notable overlap in proposed aberrant circuitry of subcortical nuclei and interactions with prefrontal cortex, linked with dysfunctional habit formation (21)
Premonitory sensations	Uncomfortable feelings that precede a tic, often perceived by patients as sensations that generate an urge to tic. When the tic is released, patients typically experience a sense of relief and resolution of the sensation. The sensations are likened to feelings of “itch” or “pressure,” and are often coupled to a bodily location related to the site of the subsequent tic (22)
Suppression	Although tics are commonly considered as involuntary acts, often patients can exercise a degree of volitional inhibition over the release of their tics. Suppression is not always successful, and usually only temporarily prevents tic release. Furthermore it is an effortful process, requiring concentration, during which the urge to tic does not diminish (23). Nevertheless, many people with TS choose to engage in tic suppression as a coping strategy in social settings (24)
Unvoluntary tics	Tics are rapid, recurrent, motor and/or phonic acts. Many people with TS report that their tics are a response to relieve uncomfortable premonitory sensations. Tics are therefore described as “unvoluntary” acts, in which a volitional decision is taken to release a tic, in order to relieve an involuntary urge (25)
Waxing and waning of tic frequency and repertoire	Tics show a characteristic “waxing and waning” profile in frequency and expression over time, with some receding and others appearing. This appears to be linked in part to stress and autonomic reactivity (26) and is also likely associated with habit forming and extinction processes (27)

Although tics are usually described as involuntary, being unwilled and unchosen, subjective reports suggest that tics follow the experience of an (involuntary) urge to move, to which the individual responds by (voluntarily) releasing the tic in order to relieve the urge (22, 29, 30). In this sense, tics can be described more accurately as “unvoluntary” (25, 31). The urges are also known as premonitory sensations, and are commonly described as uncomfortable feelings of itch or pressure that are relieved once the tic is released (22, 31, 32).

People with TS can often temporarily suppress the release of their tics, usually to avoid discomfort of social scrutiny (25, 33, 34). However, many individuals report that tic suppression is an effortful process, requiring concentration, during which the urge to release a tic does not decrease, and can indeed increase in intensity (23, 24).

## Functional Neuroanatomy of TS

In prototypical motor control, signals from cortex encoding motor plans enter cortico-striato-thalamo-cortical (CSTC) circuits via the putamen (6). A balance of excitatory and inhibitory interactions in the direct and indirect pathways, regulated by monoamines, notably dopamine, enables the basal ganglia to facilitate movement production through disinhibition of thalamic signals to the primary motor cortex (6, 35). This model of basal ganglia circuitry suggests that tics arise through dysfunction within CSTC circuits (36–39). In TS, dysregulation within these basal ganglia pathways likely compromises inhibitory striatal output (36, 38), specifically via the facilitation of direct over indirect pathway activity (40). The consequence is a disinhibition of thalamic output to motor cortex, predisposing the production of actions that were not signalled via cortico-striatal inputs, or rather, signalled only weakly.

Post-mortem data provides key insights into the basis of striatal dysfunction in TS, indicating a reduction in GABAergic interneurons of up to 50% (10–12). This is consistent with altered neural migration during neurodevelopment (10). Strikingly, in animal models, injection of the GABA antagonist bicuculline into the putamen leads to brief recurrent actions that closely resemble human tics (41, 42), reinforcing a role for dysfunctional inhibitory regulation within the putamen.

Alongside GABAergic dysfunction, abnormal dopaminergic transmission is also implicated in TS (9, 43, 44), consistent with the observed clinical utility of dopamine antagonists in reducing tics (8). Mainstay pharmacological treatments act to block D2 receptor activation (Table 1). Nevertheless, it is still unclear how dopaminergic therapies exert a beneficial effect (9, 43), beyond a broad “re-balancing” of interactions between direct and indirect pathways facilitating or preventing movement (6). Furthermore, D2 antagonists are not consistently effective in all patients (8), indicating that the role of dopamine in TS is insufficient to present a reliable treatment target for resolution of tics across heterogeneous patient populations.

In addition, cortical dysfunction, incorporating cortico-cortical and subcortical interactions, is increasingly recognised as contributing to symptoms of TS (18, 19, 39). This also makes intuitive sense in light of the broader neuropsychiatric complexity of the syndrome, encompassing tics, premonitory phenomena, and capacity for tic suppression, alongside the expression of common comorbidities.

**Figure 1:**
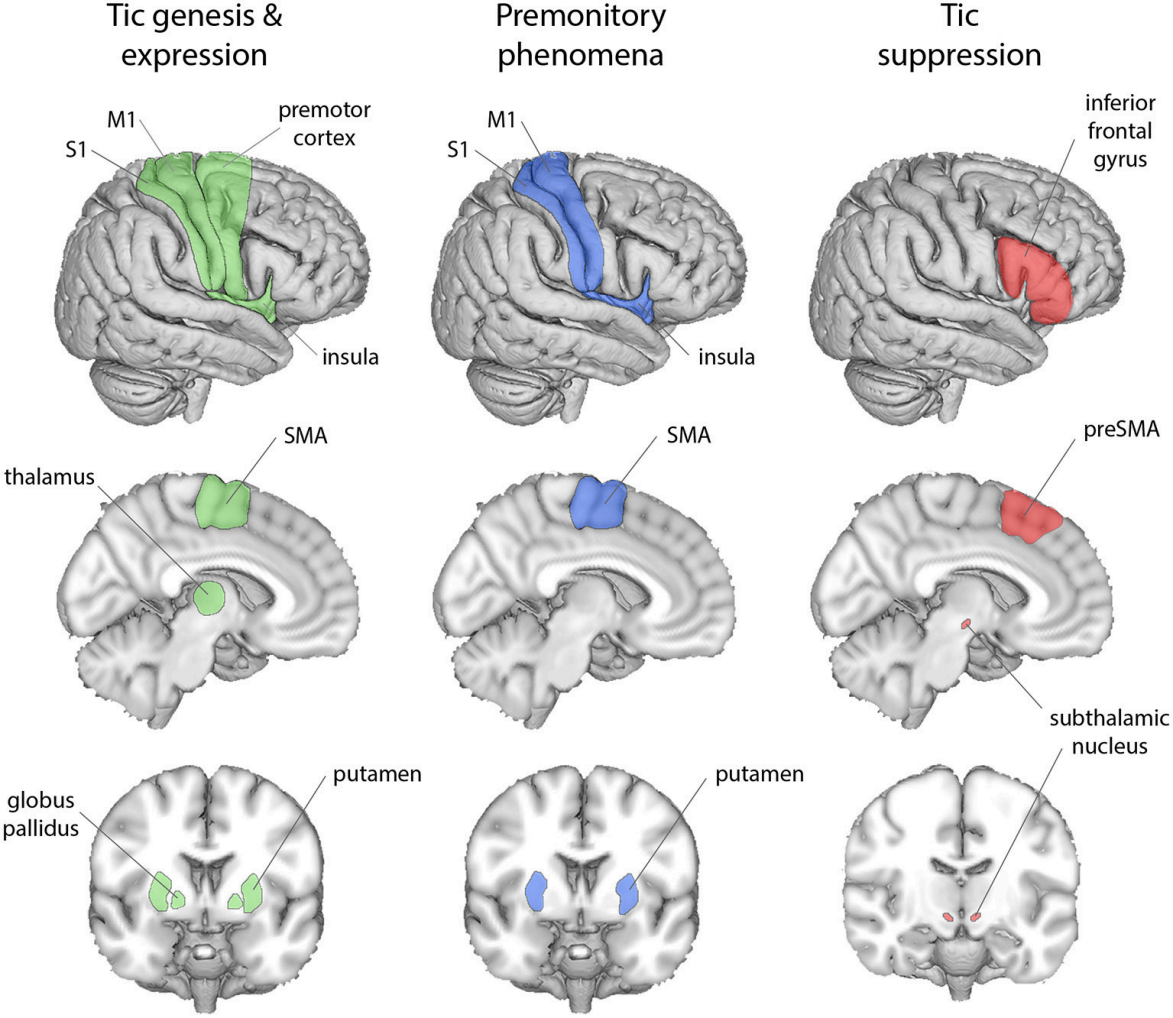
Cortical and subcortical regions associated with the phenomenological features of TS. Tic genesis and expression (green) is associated with premotor cortex, SMA, insula, M1, S1, the putamen, globus pallidus, and thalamus; premonitory sensations (blue) with the SMA, insula, M1, S1, and the putamen; tic suppression (red) with the inferior frontal gyrus, preSMA, and the subthalamic nucleus.

Functional MRI studies examining tic genesis (Figure 1) highlight specific cortico-striatal mechanisms, implicating supplementary motor area (SMA), premotor cortex, insula, sensorimotor cortex (S1 and M1), putamen, globus pallidus, and thalamus (45–47). During tic generation, cortical activity in SMA apparently precedes activity in basal ganglia, suggesting that SMA hyperactivity initiates a dynamic cascade of aberrant activity through CSTC circuits (18, 19, 48).

fMRI studies implicate insula, primary somatosensory cortex (S1) and putamen in the experience of premonitory phenomena (Figure 1) (45, 46). In addition, the volume and thickness of insula, S1, and M1 cortices correlate with premonitory phenomena (49, 50), as measured by the Premonitory Urges for Tics Scale (PUTS) (51). Moreover, the strength of resting-state functional connectivity between anterior insula and SMA also predicts PUTS scores (52).

Tic suppression engages lateral prefrontal cortex, in particular the inferior frontal gyrus (Figure 1) (53–55), and likely occurs at a later stage of signal flow through CSTC pathways, after tic initiation (56). One mechanism through which lateral prefrontal cortex activity may block the expression of a tic is through a direct modulation of subcortical inputs from regions implicated in voluntary action control such as the preSMA, notably to the subthalamic nucleus (57, 58).

## A Bayesian Account of Tics

**Figure 2:**
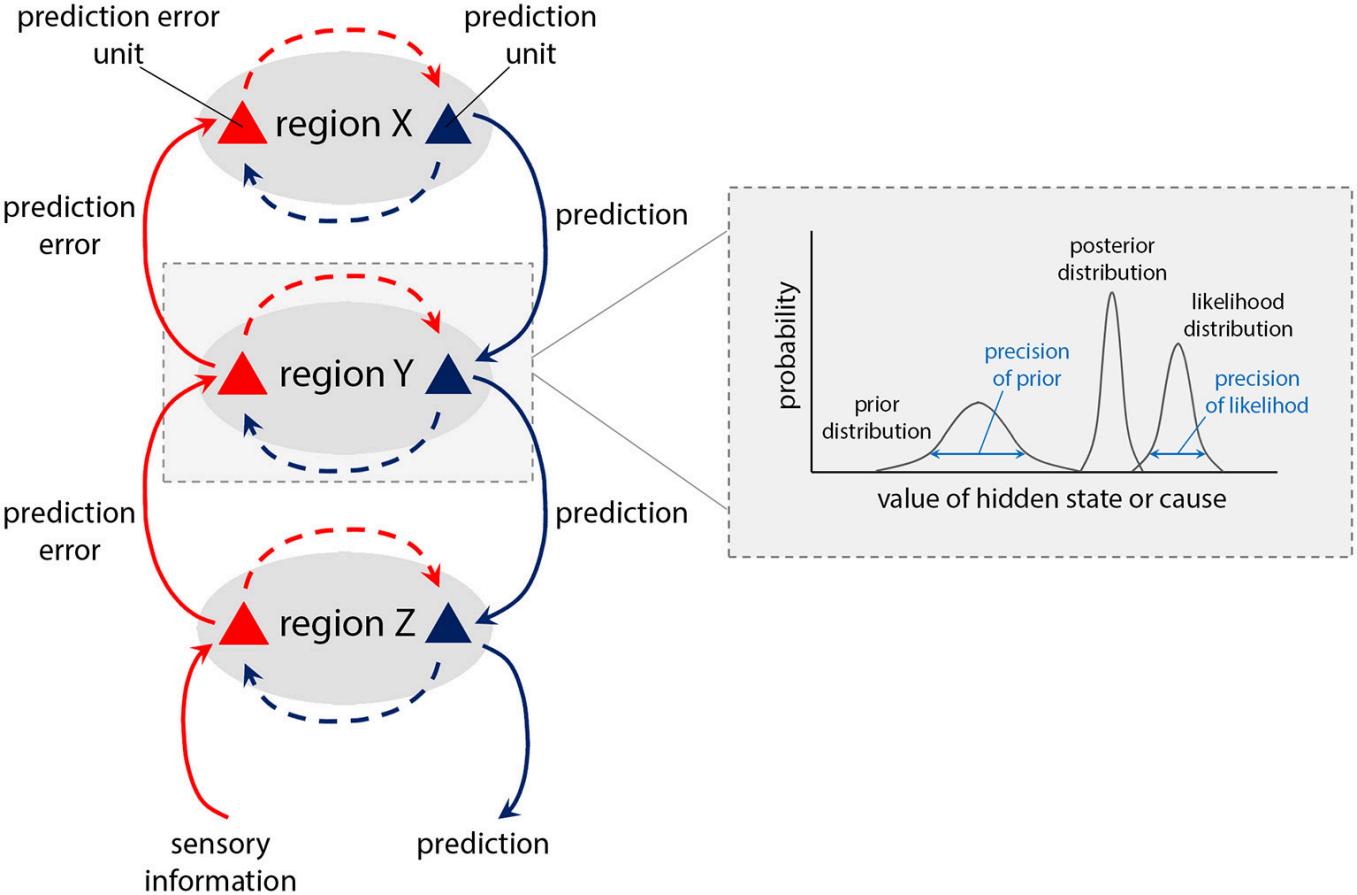
Schematic of predictive coding within a hierarchical neural system (1, 2). Descending (blue) pathways convey (*prior*) predictions about the causes of sensory inputs, while ascending (red) pathways convey sensory (likelihood) *prediction errors*, which are combined at each level through generative models encoding prior and likelihood functions, to form (Bayesian) posterior expectations. The relative contributions of prediction and prediction error are weighted according to their (expected) precision, or “reliability” (see box), which is associated with the activity of neuromodulatory systems. Such neuromodulators can typically act to attenuate or amplify the effect of a pre-synaptic neuron on its post-synaptic target, hence attenuating or amplifying descending prior or ascending prediction error signals (“post-synaptic gain”).

The modulation of interactions within CSTC networks, and their hierarchical nature, invites consideration in terms of hierarchical generative neural models of perception and behaviour, under the banner of “predictive processing” or “Bayesian brain” accounts. Broadly, these propose that perception, cognition and action result from Bayesian processes in which incoming (bottom-up) sensory signals are combined with prior (top-down) predictions, expectations, or beliefs to form the brain's “best guess” of the causes of the signal (in Bayesian terminology, the “posterior”). Operationally (Figure 2), this is achieved through the minimisation of prediction error signals, i.e., computed mismatches between predicted and actual afferent sensory signals. In predictive processing schemes, neuronal representations in higher levels of neuronal hierarchies generate predictions about representations in lower levels (hence the term “generative model”). At each level within the hierarchy, these descending predictions are compared with lower level representations to form a prediction error. This mismatch, or difference signal, is passed back up the hierarchy, to update higher representations. The recurrent exchange of signals between adjacent hierarchical levels resolves prediction error at each and every level, resulting in a hierarchically deep explanation for sensory inputs.

Importantly, prediction errors can be minimised either by updating predictions (perception) or by performing actions to change incoming sensory signals. This latter process is termed *active inference* (59, 60) and applies to both perceptual and motor hierarchies. Thus, within the motor system, high level motor predictions (priors) can cascade down into fine-grained skeletomotor predictions prescribing action sequences, to resolve proprioceptive “prediction error” signals.

The influence of prediction errors on updating priors is determined by their precision-weighting. Precision is the inverse of variance; thereby, afferent signals with high (expected) precision induce a more substantial updating of top-down predictions. The process of optimising precision-weighting has been equated with attention (61), which corresponds—neurophysiologically—to modulation of post-synaptic gain (2). In motor control, the expression of motor predictions through action (i.e., active inference) is enabled by transiently attenuating proprioceptive prediction errors, instead of updating the predictions themselves (59, 62).

Active inference within sensorimotor hierarchies has underwritten accounts of voluntary action, encompassing agency (63, 64), the interpretation of the actions of others (65, 66), and neuropsychiatric conditions, notably catatonia and functional motor disorder (67, 68). Catatonic akinesia is attributed to abnormally reduced precision of priors at high levels of the motor hierarchy (67), whereas functional motor symptoms are proposed to arise through “abnormal prior beliefs that are afforded excessive precision by attention” (68). Such proposals for increased or decreased precision at given levels of a hierarchy are underscored by behavioural and neuroimaging investigations that implicate abnormal inferential processes, evident behaviourally (see Table 2) (70, 71, 78), which also have the potential with neuroimaging to be tied to specific neuroanatomical frameworks.

**Table 2:** Experimental predictions.

**FORCE MATCHING PARADIGM**	
What is it?	This task assesses comparator processes between motor prediction and sensory outcome (69). Participants experience a force applied to their finger by a lever, and then attempt to match this force, either indirectly by using a slider with their other hand, which drives the lever, or by using their own hand directly to press the lever. Typically, in the latter case when actions are self-generated, participants tend to overcompensate by exerting larger forces. In the framework of active inference, this happens through attenuation of self-generated sensory signals during movement, which is necessary for actions to be elicited via spinal reflex arcs, even though sensory evidence indicates movement has not yet occurred (62). Actions that are not self-generated—i.e., that are similar to external events—will lack precise priors at higher levels of the motor hierarchy, and will therefore not induce sensory attenuation.
Neuropsychiatric evidence	Patients with schizophrenia (70) and functional motor disorders (71) show reduced sensory attenuation (more accurate force matching), suggesting a failure to attenuate prediction errors for actions that are self-generated, but that are perceived to be avolitional and lacking agency. This may arise through overly precise priors at intermediate levels of the motor hierarchy (68), generating actions which lack correspondingly precise priors at higher levels such as the preSMA. This entails a failure to attribute movements as self-generated, and therefore a corresponding absence of sensory attenuation.
Prediction in TS	People with TS should show more accurate force matching, due to motor predictions with high precision at intermediate levels of the motor hierarchy, without correspondingly precise priors in higher regions that are associated with attribution of actions as self-generated (such as preSMA).
**SENSORY EVOKED POTENTIALS (SEPs)**	
What are they?	SEPs (cortical signals induced by sensory nerve stimulation at effectors) can be used as neural indices of sensory attenuation (72). SEP amplitude is normally attenuated during self-generated movement when compared to rest. This process can be modulated by dopamine (73).
Neuropsychiatric evidence	In functional motor disorder (74) and dystonia (75), there is a lack of SEP attenuation, related to failure to attribute movements as self-generated.
Prediction in TS	A reduction in this SEP attenuation due to lack of precise priors for action at highest levels of the motor hierarchy; and, secondarily, that dopaminergic therapy (73) for tics may normalise this reduction.
**INTENTIONAL BINDING**	
What is it?	The intentional binding task is an informative paradigm for measuring perceptions of the relation between actions and outcomes (76). Participants make voluntary actions which are followed, after variable intervals, by a tone. Participants estimate the time of their action, or of the auditory tone. Typically, actions are perceived as occurring later when followed by a tone, and tones preceded by actions are perceived as occurring earlier: the action and the tone are thus “bound” together in time, suggesting the tone is perceived as a sensory outcome of the action. Intentional binding has been proposed to arise through a Bayesian system that predicts the sensory consequences of actions (64, 77).
Neuropsychiatric evidence	Patients with functional motor disorder show reduced intentional binding (78), suggesting increased precision of (intermediate-level) action priors, while patients with corticobasal degeneration causing symptoms of an “alien limb” show increased binding in their affected arm (79), suggesting decreased precision of action priors.
Prediction in TS	Patients with TS will show reduced intentional binding, given an increased precision of intermediate-level action priors.
**COMPUTATIONAL PSYCHIATRY**	
What is it?	A general approach in which behavioural measures or modelled parameters are integrated with functional neuroanatomical data to infer the mechanisms by which activity in neural systems generates behaviour (80).
Neuropsychiatric evidence	In corticobasal degeneration, alien limb phenomena are explained by reduced precision of action priors, illustrated by abnormal intentional binding, linked to symptom severity and dysfunctional interactions between preSMA and prefrontal cortex (79).
Prediction in TS	There are numerous opportunities to gain insights from computational psychiatry approaches: one prediction would be more accurate force matching and reduced intentional binding, associated with extent of altered functional and effective connectivity between prefrontal cortex, motor preparation areas, and basal ganglia.
**COMPUTATIONAL MODELLING**	
What is it?	Computational modelling of motor decision processes, including drift diffusion modelling, can quantify individual differences in performance of simple motor tasks (e.g., Go vs. NoGo), by parameterizing processes such as accumulation rates in favour of releasing (“go”) over withholding an action (“nogo”) (81, 82). Motor decision processes are likely to be influenced by the precision of priors and their integration in posteriors, implemented through changes in neuronal processing at specific levels within the motor hierarchy.
Prediction in TS	Parameters such as faster accumulation rates relate to the exaggerated precision of action priors in TS, and tendency to “go” over “nogo.” Combined with functional neuroimaging in a computational psychiatry approach (80) this may enable identification of specific networks and brain regions (e.g., the putamen) underpinning aberrant active inference in TS.
**DYNAMIC CAUSAL MODELLING (DCM)**	
What is it?	The functional neuroanatomical mechanisms determining motor behaviour, including the routing of neuronal signals through CSTC circuits, can be characterised using DCM (83). For example, in the field of oculomotor control, DCM has been applied to model eye movements in terms of a balance between precision of oculomotor priors, and precision of sensory attributes of eye movement targets (84). This approach confirmed that increased sensory precision—relating to attention to target—was underpinned by increased post-synaptic gain in V1 (85). Thus, the neuroanatomical insights to hierarchical interactions that DCM provides can be interpreted in terms of quantities like precision.
Prediction in TS	DCM can quantify the influence of neuromodulators on interactions within neural hierarchies, including CSTC circuits (86), and how dopaminergic therapies modulate these (87). In TS, DCM parameters will predict quantities such as precision of action priors, and modulatory effects of monoamines.

### Overly Precise Priors for Action in TS

**Figure 3:**
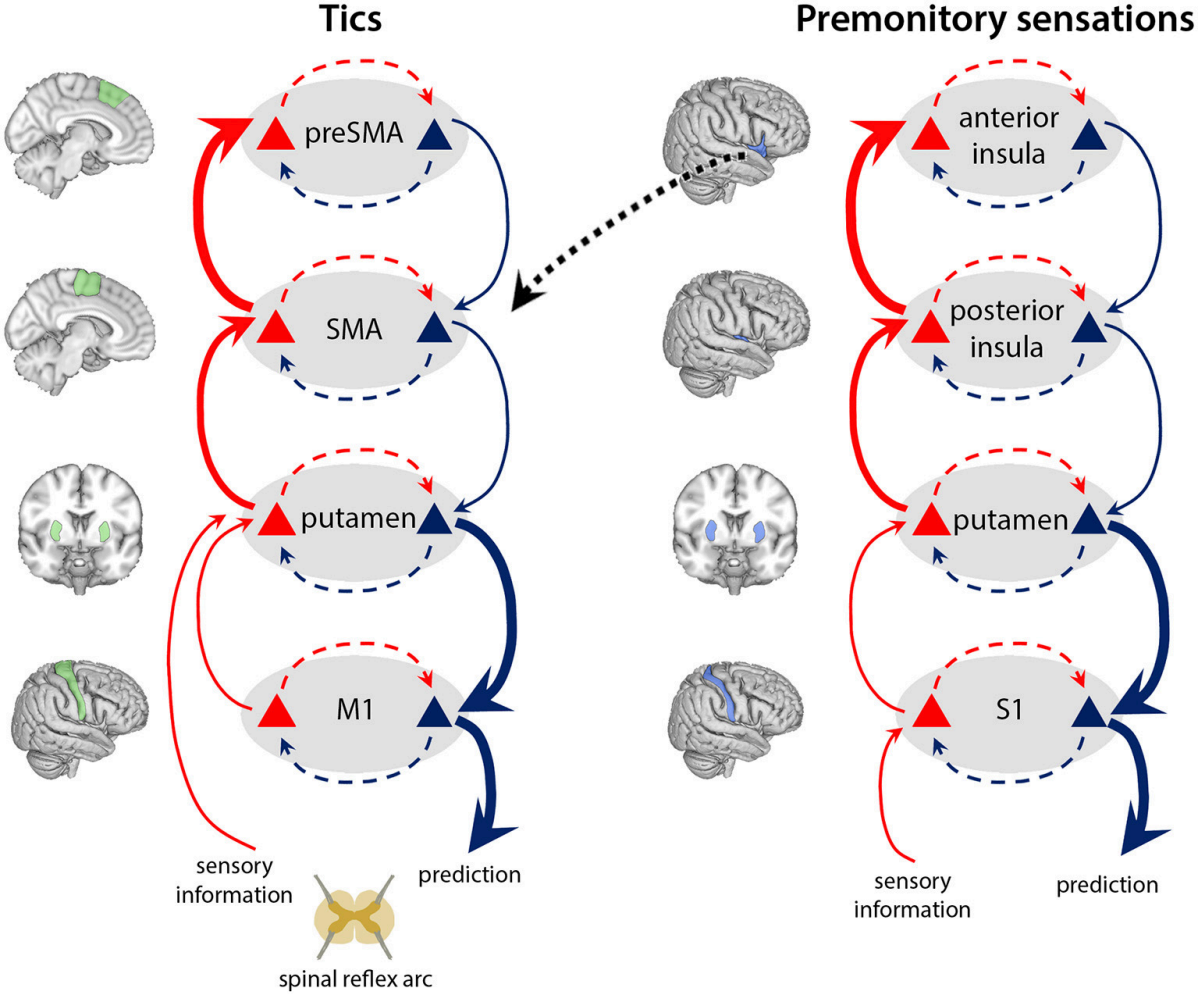
Tics and premonitory sensations arise through overly precise priors for action and sensation within the putamen. In our model of TS, increased cortical signalling from the SMA, combined with reduced regulation by inhibitory interneurons in the putamen (see Figure 4), leads to release of signals for movement to M1 with enhanced precision. These precise priors (blue) generate tics, leading to ascending prediction error signals from putamen (bold red arrows) indicating production of an action that was not predicted within high-level regions, and which is “explained away” by the preSMA ascribing an “unvoluntary” feeling to the action—that it was “somewhat intended.” Similarly, increased cortical signalling from the posterior insula, combined with reduced regulation by inhibitory interneurons in the putamen, leads to signals passing to S1 with enhanced precision. These precise priors (blue) generate bodily sensations, leading to ascending prediction error signals from putamen to anterior insula (bold red arrows) for bodily feelings that were not predicted within this higher level region, which are therefore “explained away” by the anterior insula as unexpected or “untoward” bodily feelings that require mitigating action to remove. To do so, anterior insula sends signals to midline motor regions such as the SMA (dotted black arrow): augmenting the excitatory inputs from SMA to motor putamen, further ramping up the process of tic generation. Within-layer interactions (dashed red and blue arrows) represent canonical microcircuits (88) in which superficial pyramidal neurons (red triangles) compare expectations with predictions from deep pyramidal neurons (blue): discrepancies at the highest hierarchical levels are explained away as “unvoluntary” actions (tics), and untoward bodily feelings (premonitory sensations).

**Figure 4:**
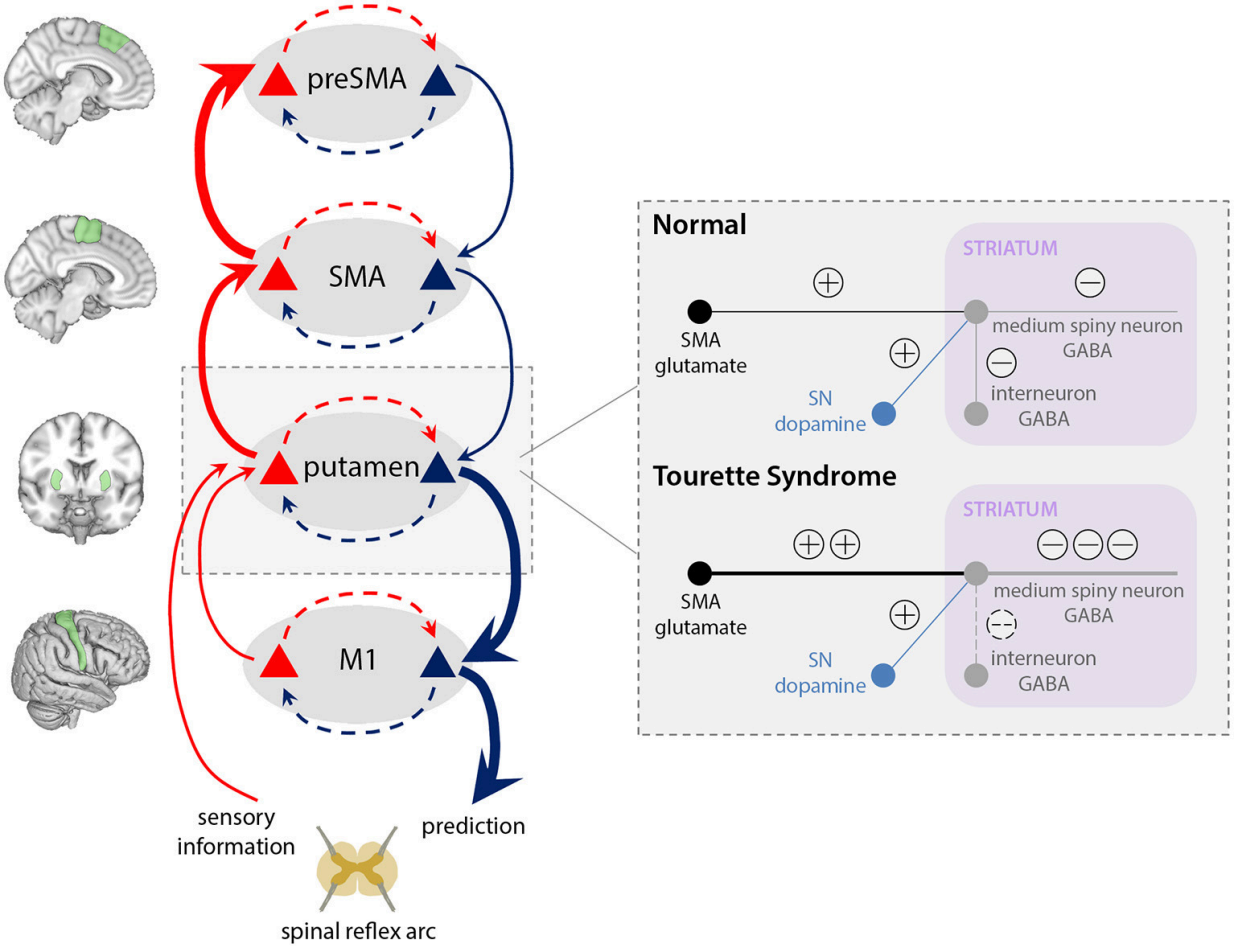
Aberrant integration of cortical inputs within the striatum in TS. Typically, glutamatergic inputs to the basal ganglia direct pathway are regulated by inhibitory interneurons within the striatum, and modulated according to dopamine release by the substantia nigra (SN). In our model of TS, increased cortical signalling from the SMA, combined with reduced regulation of the direct pathway by inhibitory interneurons, leads to increased excitation of the medium spiny striatal output neurons, which results in greater thalamic disinhibition and release of signals for movement to M1. Thus, a tic is generated, leading to ascending prediction error signals from putamen (bold red arrows) for a movement that was not predicted within preSMA, and which is “explained away” as an “unvoluntary” action.

The active inference framework provides a powerful framework for conceptualising the basis of tic generation in TS. We highlight interactions at four levels of the motor hierarchy, ascribed neuroanatomically to a CSTC circuit encompassing preSMA, SMA, basal ganglia, and primary motor cortex (Figure 3). We propose that tics arise through abnormally precise priors for action at an intermediate level of the hierarchy, specifically within the putamen, as a result of two aberrant processes. First, overactivity of the SMA [but not preSMA, (45–47)] leads to increased glutamatergic (excitatory) inputs to the putamen. Second, reduced density of GABAergic interneurons in the putamen (Figure 4) causes aberrant synaptic integration. In the direct pathway, which facilitates release of motor programs, the combined effect is a greater excitation of the medium spiny striatal output neurons, which exert an inhibitory effect over relay nuclei (Table 1). This results in thalamic disinhibition and release of signals for movement to M1, thereby increasing the probability that motor engrams with little higher-level prediction from preSMA will be released. Thus, predictions for action are generated from the putamen, and motor programs enacted that resolve the corresponding proprioceptive prediction errors through active inference, even though there is little high-level evidence for these actions; i.e., when the precision of priors for these actions within higher levels of the motor CSTC circuit is low (perhaps reflected in diminished preSMA activity).

Supporting evidence for these mechanisms comes from neuroimaging and post-mortem data: in TS, the SMA (but not the preSMA) is hyperactive during “free ticcing” (45, 46), and emerges as a site of aberrant activity, anatomically and temporally, prior to the basal ganglia (18, 19, 48). SMA outputs to the putamen are glutamatergic (89), suggesting that in TS, the excitatory input to the putamen from the SMA is amplified prior to tics. Failure to regulate this heightened input at subcortical synapses would foster the expression of overly precise predictions for action within the putamen.

GABAergic interneurons appear to regulate medium spiny neurons within the putamen by “filtering” cortical inputs, thereby controlling striatal outflow (90, 91). These interneurons preferentially target medium spiny neurons within the direct pathway that promotes the release of actions, rather than the indirect pathway (92). In TS, striatal inhibitory GABAergic interneurons are reduced by up to 50% (10–12, 93), which given their dominance in the direct pathway, would engender a relative increase in direct pathway activity over indirect (40). Furthermore, animal models also strongly implicate a role for dysfunctional inhibitory regulation within the putamen (41, 42), compatible with the notion that the precision of putamen-encoded priors for action is elevated in TS as a consequence of reduced inhibitory regulation of (predominantly direct pathway) medium spiny neurons.

The role of dopamine in tic generation remains unclear (9). Dopaminergic therapies for TS, which often have a D2 antagonist action, are not always effective (8). However, even if tonic levels of dopamine remain unaltered in TS (9)—and evidence for altered striatal dopaminergic innervation in TS remains mixed (94–96)— the high precision of predictions for action in the putamen would be sufficient when passed to M1 to elicit a tic. When dopaminergic therapies are successful in tic reduction, this may reflect a re-balancing of activity between direct and indirect pathways, by amplifying activity within the indirect pathway where D2 receptors are dominant, and so preventing relatively higher excitation of direct pathway medium spiny neurons. This could reduce generation of precise putamen priors for action.

Furthermore, we note that tics appear “habitual” in their repetitive, recurrent nature, and in the repertoire of tics unique to each individual. Dopamine likely plays a role in the establishment of tics as habitual behaviours, for example via reinforcement learning processes (44). People with TS show enhanced habit formation on laboratory tests, which furthermore correlates with structural connectivity between the sensorimotor putamen and primary motor cortex (27). This accords with the establishment within the putamen of dominant priors for specific tic actions that are likely represented on a somatotopic basis, according to somatomotor maps within the putamen (97, 98). Unless dopaminergic therapies prevent generation of these precise putamen priors, they become embedded as patterns of behaviour with high precision, namely, habits.

Although we focus on the specific CSTC motor circuit, given the histological and neuroimaging evidence for striatal dysfunction in TS, we note that motor control proceeds not only via these pathways, but also comprises interactions within direct cortical routes—such as from SMA to M1 (99)—and extracortical routes such as via cerebellar circuitry (7). While, in our model, striatal dysfunction within CSTC pathways represents a core substrate for generation of precise priors for action in TS, this does not preclude the possibility that such active inference processes also contribute to tics via these alternative routes. To better understand which motor networks, and precisely where within those networks, dysfunctional active inference processes may arise, techniques such as computational psychiatry approaches can be applied to disentangle component circuitry (see Table 2). Fundamentally, active inference proposes that actions are generated by predictions that “are communicated through the firing rate modulation of descending efferents” (100), which may be accomplished through increased precision for action within the CSTC pathway we highlight, or via other routes to primary motor cortex.

### “Unvoluntary” Tics

A key phenomenological feature of tics is their “unvoluntary” nature (25). We suggest this arises through ascending prediction errors from the putamen (bold red arrows, Figure 3), signalling production of an act for which a higher-level goal or “intention” was not encoded in the preSMA. An “unvoluntary” experience occurs through these prediction errors being “explained away” by the preSMA as the action having been “somewhat intended.” This notion accords with evidence that the preSMA has a role in attending to one's own intentions (101, 102), and is associated with feelings of intention to move (103).

### Premonitory Phenomena

Premonitory phenomena are experienced by the majority of adolescents and adults with TS (22, 29, 32), albeit not alongside every tic. We propose these emerge through dysfunctional interactions at four key “bodily representation” levels of a CSTC hierarchy, mirroring the hierarchical motor circuit relating to tics: anterior insula, posterior insula, basal ganglia, and primary somatosensory cortex (Figure 4). As with increased precision of prediction at intermediate levels of the motor hierarchy, abnormally increased precision of prediction within the somatosensory region of the putamen likely underpins premonitory bodily sensations. These abnormally precise predictions pass to S1, eliciting sensations as they overwhelm sensory inputs. Ascending signals reporting these sensations then progress through the hierarchy to bodily perception regions, notably the posterior and anterior insula. At the level of anterior insula there are substantial prediction errors to resolve, since these sensations were not predicted by insula activity, relating to momentary interoceptive representations of bodily state. As a result of “explaining away” these high-level prediction errors, an individual experiences “untoward” bodily feelings, which underpin the occurrence of premonitory phenomena.

These processes parallel the “explaining away” of ascending movement-related prediction error signals as “unvoluntary” tics by the preSMA (Figure 3). In this case, the ascending prediction error from putamen, reflecting unpredicted feelings, upon reaching a higher level region may be “explained away” by the anterior insula as a “sensory symptom” (Figure 4). It is notable that interoceptive accuracy, which is associated with anterior insula function (104), is reduced in people with TS, and correlates with severity of premonitory urges (105). This accords with the hypothesis that interoceptive prediction errors, arising within the anterior insula, contribute to the experience of premonitory sensations as unexpected bodily feelings that require mitigating action to remove.

The two processes underpinning premonitory sensations and tics are also likely causally linked (40). Fulfilling a proposed role in maintaining homeostatic integrity (106–110), the anterior insula may trigger mitigating action for removal of such unexpected sensory symptoms, via signals to midline motor regions, such as the SMA (Figure 3, dotted black arrow). From the SMA, tic generation may then arise through an increase in glutamatergic inputs to putamen, and failure to regulate these predictions in the motor hierarchy as described above. With the production of mitigating action, via feedback signals to insula (111) homeostatic balance is reinstated, and a sense of relief is experienced with the expunging of the sensory phenomena (29). Interestingly, severity of premonitory sensations is predicted by the strength of functional connectivity between anterior insula and the SMA (52). Furthermore, this link between anterior insula and SMA hyperactivity may underpin the observed increases in tic severity that can often occur under psychological stress (112), particularly in social contexts, with the associated effects of scrutiny and stigma (113): when people with TS view emotional faces, a hyperactive insula is associated with increases in functional connectivity to cortical motor regions (114). Furthermore, severity of premonitory sensations is predicted by functional connectivity between the insula, and the SMA, under these conditions (114). This suggests that a hyperactive insula may trigger mitigating action, in the form of tics, for removal of uncomfortable bodily feelings that underpin emotional experiences, such as stress and anxiety (115).

Evidence for these mechanisms again comes from neuroimaging and post-mortem data: in TS, neuroimaging has identified the (posterior) insula as a consistent site of abnormality, where increased activity precedes tics (45, 46). Moreover, decreased insular GABA_A_ receptor density (14), and decreased cortical volume are observed in TS, the latter associated with severity of premonitory phenomena (50). Together, putative hyperactivity of the (posterior) insula in TS leads to increased excitatory inputs to the somatosensory regions of the putamen.

Representations of bodily state within the mid and posterior insula are organised in a somato- and viscero-topic manner, maintained in projections to the striatum (97, 116). The somatosensory region of the striatum largely overlaps with the area of the putamen subserving motor function (98). As with motor signals, somato- and viscero-sensory inputs are likely to be subject to a similar impact of reduced GABAergic interneurons within the putamen, which results in a failure to filter incoming signals and regulate striatal outflow.

The role of a subcortical somatomotor map in TS is reinforced by the tight coupling of bodily location of premonitory sensation and site of tic emergence (22). In addition, observations that premonitory phenomena are most commonly experienced preceding facial and neck tics (22) may relate to the specific neuroanatomical site within a putamen somatomotor map in which interneuron and synaptic integration dysfunction is most marked, the location of which will likely vary between individuals. Finally, successful tic suppression follows a somatotopic distribution according to bodily locations that “tic the least” (56), suggesting that precise priors become embedded and underpin habitual tics according to somatotopic maps. The putamen is thus implicated as the site of overly precise priors for both tics and premonitory phenomena, in contrast to cortical regions (e.g., the anterior insula) that do not support such tightly mapped sensorimotor representations. As with motor circuitry, there are further pathways supporting somato- and viscero-sensory function in which dysfunctional active inference processing may occur, beyond the proposed overly precise priors within CSTC circuitry: for example, there are direct cortical routes between insula and S2 (117), which may contribute to the generation of premonitory sensations. However, in light of the histological and neuroimaging evidence for insula and striatal dysfunction underpinning premonitory sensations, it is likely that CSTC circuitry plays a key role.

Within the CSTC circuits, while histological evidence suggests the presence of monosynaptic inputs from insula to the striatum (97, 117), to date, no studies have examined the putative reverse connections that would mediate ascending prediction errors from the striatum to insula. Although the histological work for such monosynaptic connections is yet to be performed, given the existing evidence for hierarchical feedback loops in parallel CSTC systems (98), such as the motor pathways, such routes seem anatomically plausible. Furthermore, while active inference schemes assume that ascending connections in a hierarchy carry prediction error signals, it is not a principled requirement of the framework that that these be monosynaptic.

Although up to 60% of tics are associated with premonitory phenomena (22), the fact that some tics do not have strong premonitions suggests such bodily feelings are likely not a prerequisite for triggering of tics. However, when premonitory phenomena occur, they may further ramp up the likelihood of tic generation through insula signalling into the SMA, augmenting the excitatory inputs from SMA to motor putamen, making tics even more likely. This process likely also underpins the observation that tics can be exacerbated by psychological stress (112), including social contexts potentially associated with scrutiny and stigma (114).

### Tic Suppression

Many individuals with TS are able to suppress tics, albeit sometimes for only a short period of time and with considerable effort (24). Tic suppression likely proceeds via the hyperdirect pathway (118) from cortex to the STN. The STN is proposed to function as a rapid “brake” for pausing motor output when remedial action is required, until a more appropriate line of action is determined (57). The STN receives cortical inputs via the hyperdirect pathway from lateral prefrontal cortex, in particular, the inferior frontal gyrus (57, 119), which exerts a modulatory effect on signals from cortical motor preparation areas, such as the preSMA, on the STN, to achieve inhibitory control (58). We propose that the volitional and effortful tic suppression reported by individuals with TS occurs via recruitment of lateral prefrontal regions to invoke hyperdirect pathway activation, by modulating cortical inputs from regions such as the preSMA to subcortical nuclei, and specifically, the STN (Figure 1). In line with this, fMRI studies of tic suppression implicate the inferior frontal gyrus, where the magnitude of activation predicts effective tic suppression (53, 55). More broadly, the preSMA is strongly associated with volitional control of action, including both decisions to move, and to not move (120–122). This suggests that during tic suppression, the preSMA may signal an instruction for volitional suppression of movement to the STN, which is augmented at the STN input synapse by subcortical signals from the inferior frontal gyrus (58, 86).

In the context of active inference, this prefrontal mechanism may correspond to increasing the precision-weighting of proprioceptive prediction errors (through attention), transiently diminishing the influence of high-precision priors for tic generation. However, tic suppression is not always successful: this mechanism seems only to pause and defer outflow to the thalamus, without resolving the imbalance within the striatum of excitatory inputs from the SMA, and poor regulation by inhibitory interneurons.

Suppression success is related to tic frequency: tics that are least common hold the highest chance of success (56). Tics that have become habitual, through high precision embedded with repeated performance, may still be generated. However, for less habitual tics, where precision of their striatal signals is lower, the pausing of basal ganglia outputs with STN activation may buy sufficient time for blocking the predictions for action from the putamen, perhaps via the indirect pathway.

A key feature of tic suppression success is the somatotopic distribution, with tics easiest to suppress in body parts that “tic the least”: implying suppression occurs after activity relating to generation of tics has passed through a somatomotor filter (56), as may be present within somatomotor putamen. Correspondingly, the face, larynx and upper body are the most commonly and severely affected body parts by tic disorders, consistent with these regions having the largest representation within cortico-striatal somatomotor circuits. Suppression thus follows generation of precise priors for action within somatomotor putamen regions, through presumably prefrontal-driven pausing of basal ganglia outflow by the STN at this later stage (56).

Often the urge to release a tic does not decrease while patients engage in effortful suppression (24). This is consistent with the proposal, discussed above, that cortical drive to the STN may pause outflow from the basal ganglia (123), but cannot reduce the high precision of the striatal signal, linked to motor programs and (often) premonitory sensations. The precise sensory and motor predictions therefore continue to generate ascending prediction errors until the tic is released, or, until precision of cancellation signals within the indirect motor pathway become higher, generated for example by parallel cortical inputs to prevent actions (124), or by pallidal signals (123). Alternatively, the individual may perform a substitute action that meets the high prior for action precision (such as singing in place of a phonic tic).

### Additional Features: Behavioural Strategies, “Waxing and Waning” of Tics, and Developmental Changes

Replacement of tics with alternative actions, or distraction with an engaging activity, are common strategies employed by individuals with TS and form core strands of behavioural therapies such as Habit Reversal Therapy and Comprehensive Behavioural Intervention for Tics (125). The anecdotal experiences of patients who find musical performance temporarily reduces tic frequency has been well-documented (126). We suggest these approaches are effectual through two different mechanisms. First, by meeting the highly precise prior for movement when producing alternative actions or musical performance. Second, through attenuation of precise priors for action, by switching attention to an alternative program of goal-directed behaviour, as in the infamous case of the surgeon with tics, “CB” (127).

Alongside the embedding of certain tics as habits (see above), many tics show a classic “waxing and waning” profile, with the frequency and expression of an individual's tic repertoire increasing and decreasing over a period of weeks and months (128). While the driving factors remain poorly understood, it is likely that both autonomic tone and autonomic reactivity contribute (26), alongside habit formation and extinction processes associated with the striatum (27). Changes in cortical excitability accompany states of heightened autonomic arousal, and could thereby increase the precision of priors for action at the cortical input synapse to the putamen, correspondingly, raised sympathetic (electrodermal) activity is associated with an increased likelihood of tic generation (129).

Following the onset of tics in childhood and adolescence, a common profile of TS is one which tic severity gradually lessens into adulthood, although individual differences are marked and in some people, tics may worsen (130). In the context of a Bayesian account of tics, such developmental changes may relate to synaptic pruning processes that occur through adolescence (131), which could result in reduced cortical excitability in the SMA, leading over time to lower precision of priors for action at the putamen input synapse. In particular, within-region changes to tonic tone via plastic changes to GABAergic interneuron density in, for example, SMA, may also be driven by compensatory processes in those individuals who experience a reduction in tic severity (13, 34).

## Experimental Predictions

This Bayesian account of tics motivates a number of novel experimental approaches, based on manipulating motor predictions and modelling motor processes in hierarchical inferential networks. Paradigms that test active inference processes such as sensory attenuation, complemented by model-driven neuroimaging methods (80, 132), hold promise for detailing the neuropathological mechanisms underpinning tics within CSTC hierarchies (Table 2).

To date, no investigations have applied sensory attenuation approaches (62), such as the force matching paradigm or measuring sensory evoked potentials, in people with TS. However, in the related condition of functional motor disorder, in which individuals experience symptoms such as weakness or tremor without an organic cause, these measures have provided sensitive indicators of increased precision of priors for action at an intermediate level of the motor CSTC circuit hierarchy (68, 71, 74). These complementary insights from functional motor disorder suggest that people with TS will show more accurate force matching, and a reduction in SEP attenuation, due to motor predictions with high precision at intermediate levels of the motor hierarchy, and lack of correspondingly precise priors for action at highest levels (i.e., preSMA).

Beyond sensory attenuation paradigms, tests that measure perceptions of causal coupling between actions and outcomes, such as the intentional binding paradigm (76), can also support the interpretation of altered feelings of agency in the context of a Bayesian system that predicts the sensory consequences of actions (64). Such tests predict that people with TS will show reduced action binding, with a perceptual shift to later temporal estimation of their own movements from the onset of their act, consistent with a reduced sense of agency, and thereby implicating high precision for action within intermediate levels of a motor hierarchy, as is the case for functional motor disorder (78). Research in this line would speak to fascinating questions about the neural basis for phenomenology of voluntary action in individuals with TS, in particular, when leveraged in combination with computational psychiatry neuroimaging approaches (80, 133). Such research, however, should bear in mind that intentional binding effects may reflect general multisensory causal binding, without necessarily directly reflecting intention or agency *per se* (77), and that multisensory perception, as assessed by the rubber hand illusion, is altered in TS (134), in addition to any changes in feelings of agency.

A computational psychiatry approach, broadly, aims to test model-based hypotheses, by applying behavioural parameters (often estimated trial-by-trial) to neural data: for example, increased action binding in the intentional binding paradigm is seen to predict severity of alien limb phenomena in alien limb syndrome, according to dysfunctional interactions between preSMA and prefrontal cortex, implying reduced precision of voluntary action priors within preSMA (79). By combining functional neuroanatomical data and relevant behavioural parameters, a computational psychiatry approach enables mechanistic delineation of the bridge between a neural system and observed behaviour, enabling hypothesis-driven testing of generative models.

In addition, more specific modelling approaches such as drift diffusion indices of motor behaviour (82) and Dynamic Causal Modelling of neuroimaging data (86) will permit mechanistic insights into the operation of inferential hierarchical neural systems (Table 2).

## Future Directions

Beyond experimental evidence explicitly tied to inferential action processing in people with TS, further work on the neuroanatomy relating to the core symptoms of tics and premonitory sensations, and understanding of the process of tic suppression, are key directions for future investigation.

Although the post-mortem histology to date points towards a reduction in GABAergic interneurons (and of other types) in the putamen, sample sizes are typically small (10–12). Greater histological work, extending beyond the subcortex to key cortical regions such as the SMA and insula, is crucial for understanding the precise nature of CSTC network alterations in TS and integration of signals within and between levels of the hierarchy.

Alongside histological work, valuable evidence on the neurochemical balance in such regions is facilitated by advances in higher field MRI that permit quantification of tonic levels of key molecules such as a glutamate and GABA via magnetic resonance spectroscopy (13, 135). However, these techniques are yet to be applied at high field in TS in relevant subcortical regions, such as the putamen.

In addition to “pure TS” samples, it is also important to delineate the neuroanatomical overlap with the commonly comorbid conditions ADHD and OCD. This neurodevelopmental triad all feature dysfunction within fronto-striatal networks (136) and are associated with particular genetic profiles (137). The coexistence of one, two, or three of the conditions within an individual likely relates in part to the location and anatomical extent within the striatum of dysfunctional circuitry, such as interneuron density, and how this influences cortical afferent and efferent signalling. This implies that parallel hierarchical inferential schemes for attention deficit and obsessive compulsive symptoms are also legitimate models underpinning the “TS triad.” Autistic spectrum disorders also show notable comorbidity with TS (113). Some models have proposed that increased sensory precision in perception and cognition may underpin ASD symptoms (138), suggesting that even within one individual, alterations to precision of priors, or sensory likelihoods, may vary differentially across sensory, cognitive, and motor domains. In addition to the common comorbidities, anxiety often presents alongside TS symptoms, although not all patients with tics meet diagnostic criteria for anxiety disorders, highlighting further the heterogeneity of patients' individual clinical experience (113). Narrowing down the specific neuropathological alterations relevant to given sets of symptoms will be helpfully informed by computational psychiatry neuroimaging approaches, in which particular behavioural parameters can be linked to precise neural network dysfunctions (139).

Looking towards therapeutic strategies for TS, detailing the neuropathological mechanisms that underpin tics, premonitory phenomena, and associated comorbid symptoms at this systems level will helpfully inform the application and optimisation of novel treatment approaches, such as Deep Brain Stimulation (140) and biofeedback therapy (141).

## Conclusions

The symptoms of TS arise through dysfunctional interactions within somatomotor hierarchies. We have conceptualised these interactions within a Bayesian active inference framework, wherein actions are elicited through fulfilment of motor predictions. In this view, tics and premonitory sensations arise through overly precise priors that emerge at intermediate hierarchical levels, specifically within somatomotor regions of the putamen, due to aberrant striatal synaptic integration of cortical inputs. These processes generate predictions for action and sensation, which engender tics. They also lead to ascending prediction errors for movements and feelings that were unpredicted at higher levels within cortico-striato-thalamo-cortical (CSTC) hierarchies, and which become explained away as “unvoluntary” actions and premonitory phenomena respectively. Experimental manipulations of motor predictions, combined with computational modelling and neuroimaging, can test predictions emerging from this account and shed new light on the neurocognitive mechanisms underlying TS, its symptoms, and the efficacy of potential interventions.

## Author Contributions

CR prepared the manuscript. HC and AS reviewed the manuscript. All authors contributed to and approved the final manuscript.

### Conflict of Interest Statement

The authors declare that the research was conducted in the absence of any commercial or financial relationships that could be construed as a potential conflict of interest.
